# Medication utilization patterns among type 2 diabetes patients initiating Exenatide BID or insulin glargine: a retrospective database study

**DOI:** 10.1186/1472-6823-13-20

**Published:** 2013-06-22

**Authors:** Manjiri Pawaskar, Machaon Bonafede, Barbara Johnson, Robert Fowler, Gregory Lenhart, Byron Hoogwerf

**Affiliations:** 1 Eli Lilly and Company, Indianapolis, IN, USA; 2 Truven Health Analytics (formerly the Healthcare Business of Thomson Reuters), 77 Rowell Road, Brentwood, NH 03833, USA

## Abstract

**Background:**

Type 2 diabetes is a common and costly illness, associated with significant morbidity and mortality. Despite this, there is relatively little information on the ‘real-world’ medication utilization patterns for patients with type 2 diabetes initiating exenatide BID or glargine. The objective of this study was to evaluate the ‘real-world’ medication utilization patterns in patients with type 2 diabetes treated with exenatide BID (exenatide) versus insulin glargine (glargine).

**Methods:**

Adult patients( ≥18 years of age) with type 2 diabetes who were new initiators of exenatide or glargine from October 1, 2006 through March 31, 2008 with continuous enrollment for the 12 months pre- and 18 months post-index period were selected from the MarketScan® Commercial and Medicare Databases. To control for selection bias, propensity score matching was used to complete a 1:1 match of glargine to exenatide patients. Key study outcomes (including the likelihood of overall treatment modification, discontinuation, switching, or intensification) were analyzed using survival analysis.

**Results:**

A total of 9,197 exenatide- and 4,499 glargine-treated patients were selected. Propensity score matching resulted in 3,774 matched pairs with a mean age of 57 years and a mean Deyo Charlson Comorbidity Index score of 1.6; 54% of patients were males. The 18-month treatment intensification rates were 15.9% and 26.0% (p < 0.0001) and the discontinuation rates were 38.3% and 40.0% (p = 0.14) for exenatide and glargine, respectively. Alternatively, 14.9% of exenatide-treated patients switched therapies, compared to 10.0% of glargine-treated patients (p < 0.0001). Overall, glargine-treated patients were more likely to modify their treatment [hazard ratio (HR) = 1.33, p < 0.0001] with shorter mean time on treatment until modification (123 vs. 159 days, p < 0.0001). Compared to exenatide-treated patients, glargine-treated patients were more likely to discontinue [hazard ratio (HR) = 1.25, p < 0.0001] or intensify therapy (HR = 1.72, p < 0.0001) but less likely to switch (HR = 0.71, p < 0.0001) the index therapy.

**Conclusions:**

Patients treated for type 2 diabetes with exenatide BID or insulin glargine differ in their adherence to therapy. Exenatide-treated patients were less likely to discontinue or modify treatment but more likely to switch therapy compared to glargine-treated patients.

## Background

Diabetes is a leading cause of morbidity and mortality in the United States [1] and is a key independent risk factor for a number of microvascular and macrovascular diseases. According to the American Diabetes Association (ADA) and European Association for the Study of Diabetes (EASD) Consensus Statement, type 2 diabetes is characterized by progressive loss of beta cell function and a corresponding need for progressive and early addition of glucose lowering strategies [2]. Achieving and maintaining glycemic control as a primary treatment goal for the management of type 2 diabetes is challenging.

Several studies have examined glucose lowering treatment patterns for type 2 diabetes, showing suboptimal use and poor adherence/persistency with current therapies [3-5]. Failure to continue their glucose lowering medications may be associated with adverse outcomes including both clinical as well as economics consequences. The Third National Health and Nutrition Examination Survey found that many patients with type 2 diabetes fail to achieve optimal glycemic control and are at increased risk of developing complications [6]. A review article by Cramer reported that poor adherence to glucose lowering medications may lead to suboptimal glycemic control and also be associated with increased healthcare resource utilization and costs (e.g. due to hospitalizations, temporary or permanent disability because of diabetes related complications) [7]. Stuart et al. reported a positive relationship between persistency to glucose lowering medication and decreased healthcare costs and lower hospitalization rates [8].

Inadequate glycemic control may signal the need for assessment and treatment modification. In order to improve patient outcomes, it is essential to understand the medication use behavior of comparatively newer therapies such as exenatide BID (exenatide), a glucagon like peptide 1 (GLP-1) receptor agonist and insulin glargine (glargine) for the treatment of type 2 diabetes. Although, the clinical efficacy of both exenatide and glargine are well established, there is little information available on the ‘real-world’ economic and medication utilization patterns of these therapies. Hence, drug utilization studies using administrative pharmacy claims data can provide useful insights into the prescribing patterns and patient medication-taking behavior for exenatide and glargine in usual-care settings.

The goal of this retrospective observational study was to understand more clearly the progression of treatment patterns for patients initiating exenatide or glargine using an evaluation of the rates, timing and likelihood of treatment modification. This information may be particularly useful for health plans and providers planning interventions aimed at improving patient glycemic control and other health outcomes.

## Methods

### Study design

A retrospective, observational study was conducted, using a large managed care claims database that comprised of patients with type 2 diabetes initiating exenatide or glargine treatment. Data were derived from the MarketScan® Commercial Claims and Encounters (Commercial) Database and the Medicare Supplemental and Coordination of Benefits (COB) (Medicare) Database.

### Patient selection

Patients aged 18 years and older with at least one prescription claim for exenatide or glargine from October 1, 2006 through March 31, 2008 were selected and screened for continuous eligibility for the 12 months pre- and 18 months post-index period. The index date was defined as the date of the first exenatide or glargine prescription in the above time window.

To ensure the inclusion of only patients with type 2 diabetes, patients were excluded if they had a medical claim with a diagnosis code for gestational diabetes or type 1 diabetes. Patients with diagnosis code for chronic kidney disease, Cushing syndrome, acromegaly, a gastric bypass or banding procedure or occurrence of ≥2 prescription claims for a systemically administered glucocorticoid were also excluded. Patients who lacked a pharmacy benefit or transitioned from the Commercial to Medicare database during the pre- and post-index were also excluded in order to appropriately track their medication use patterns. Patients with any of the following prescriptions in the pre-index period were excluded: pramlintide, sitagliptin, exenatide or insulin (including glargine). Study diagnosis, procedure, and drug codes are available from the authors.

### Outcomes measurement

#### Variables

Demographic variables included gender, age, geographic region, and health plan type. Clinical variables measured included the presence of diabetes related microvascular complications (diabetic retinopathy and macular edema; diabetic neuropathy; amputation and ulceration; renal disease) and macrovascular complications (myocardial infarction; ischemic heart disease; congestive heart failure; peripheral vascular disease; cerebrovascular disease) and presence of common comorbidities (hypertension; dyslipidemia; depression; obesity; hypoglycemia). The Deyo Charlson Comorbidity index (CCI) [9] score was calculated to measure the severity of comorbid conditions. The study also captured the provider specialty, pre-index use of medications, average number of prescriptions and average number of drug classes administered by patients.

#### Treatment modification

**Treatment modification** in the matched cohorts was defined as the first event of change in the index medication in the 18 months post-index period and was classified further into 3 types: intensification, switching or discontinuation of the index medication.

**Discontinuation** was defined as a 90-day gap following the end of the days’ supply of the previous prescription claim for an index medication, ***without*** any other prescription for a non-index glucose-lowering therapy in that 90-day gap. **Switching** was defined as a prescription claim for a non-index glucose lowering medication without a refill of the index medication in the 90 days following the end of the days’ supply of the previous claim for the index prescription, ***with*** at least one refill of the new glucose lowering medication in the 90 days. **Intensification** was defined as the addition of a non-index glucose-lowering medication; continuation of a pre-index medication does not qualify as intensification. Additions were indicated by a non-index glucose-lowering medication prescription with overlap of its days’ supply with that of the index medication, followed by refills of the index medication and the added medication in the 90 days following the end of the days’ supply specific to each medication. Exenatide intensification was indicated by the addition of a new glucose-lowering medication while glargine intensification was indicated by the addition of a new glucose-lowering medication or by a dose increase of at least 100%. The definition for intensification was modified for glargine as glargine dose is escalated upwards before adding any new medication. Dose was calculated for each glargine prescription by dividing the total insulin units dispensed for a prescription by the number of elapsed days between that dispense date and the next, as the actual dose of insulin prescribed is not recorded in the claims database.

**Treatment durability** was functionally defined as the absence of treatment modification and is measured as the time on index treatment without treatment modification (such as switching, intensifying, or discontinuing).

### Analysis

#### Propensity score matching process

Because this was an observational study and randomization of patients is not possible, propensity score matching was used to ensure a similar distribution of specific characteristics between the exenatide and glargine patients. Propensity score matching reduces selection bias that might arise when comparing two different treatment options. Variables used in the 1:1 matching included the baseline characteristics listed in Table 1: gender, age, geographic region, health plan type, CCI score, diabetes related complications, other common comorbidities, physician specialty, use of glucose lowering medications (not including blood glucose self-monitoring devices) and cardiovascular medications and healthcare resource use in the pre-index period.

**Table 1:** Baseline demographic and clinical characteristics of patients before and after matching

**Characteristic**	**Pre-match**				**P value**	**Post-match**^**1**^				
	**Patients treated with**					**Patients treated with**				
		**Exenatide BID**		**Insulin glargine**			**Exenatide BID**		**Insulin glargine**	
		**N = 9,197**		**N = 4,499**			**N = 3,774**		**N = 3,774**	
	**N/Mean**	**%/SD**	**N/Mean**	**%/SD**		**N/Mean**	**%/SD**	**N/Mean**	**%/SD**	**Std. diff.**
**Age: mean (years)**	54	10	59	12	< 0.0001	57	10	57	12	7.8
**Age: group**					< 0.0001					
18-34	267	2.9%	72	1.6%		71	1.9%	66	1.7%	1.0
35-44	1,183	12.9%	397	8.8%		368	9.8%	381	10.1%	1.2
45-54	3,135	34.1%	1,146	25.5%		1,077	28.5%	1,073	28.4%	0.2
55-64	3,307	36.0%	1,382	30.7%		1,289	34.2%	1,272	33.7%	1.0
65+	1,305	14.2%	1,502	33.4%		969	25.7%	982	26.0%	0.8
**Sex: female**	5,359	58.3%	1,948	43.3%	< 0.0001	1,720	45.6%	1,726	45.7%	0.3
**Diabetes complications**										
Microvascular	1,521	16.5%	1,109	24.6%	< 0.0001	769	20.4%	766	20.3%	0.2
Macrovascular	1,530	16.6%	1,349	30.0%	< 0.0001	879	23.3%	906	24.0%	1.7
**Deyo charlson Comorbidity index**	1.4	1.1	1.8	1.5	< 0.0001	1.6	1.3	1.6	1.3	0.1
**Medication claims**	41.2	25.1	40.8	27.2	0.37	41.0	24.9	40.4	26.9	2.2
**Medication classes**	9.9	4.4	9.9	4.7	0.60	9.7	4.2	9.8	4.6	1.5
**Pre-index inpatient admissions**	815	8.9%	952	21.2%	< 0.0001	530	14.0%	543	14.4%	1.0
**Pre-index total healthcare costs**	$9,749	$12,251	$14,536	$31,763	< 0.0001	$11,194	$15,747	$11,245	$18,254	0.3
**Physician specialty**					< 0.0001					
Primary care	6,346	69.0%	3,170	70.5%		2,722	72.1%	2,699	71.5%	1.4
Endocrinology	1,168	12.7%	210	4.7%		204	5.4%	198	5.2%	0.7
Other specialist	1,016	11.0%	680	15.1%		524	13.9%	529	14.0%	0.4
Missing/unknown	667	7.3%	439	9.8%		324	8.6%	348	9.2%	2.2
**Treatment pre-Index**										
Glucose lowering	9,188	99.9%	4,402	97.8%	< 0.0001	3,765	99.8%	3,766	99.8%	0.6
Biguanides (metformin)	6,761	73.5%	2,940	65.3%	< 0.0001	2,624	69.5%	2,626	69.6%	0.1
Sulfonylureas	4,252	46.2%	3,011	66.9%	< 0.0001	2,445	64.8%	2,428	64.3%	0.9
Meglitinides	466	5.1%	259	5.8%	0.090	212	5.6%	216	5.7%	0.5
Thiazolidinediones	4,688	51.0%	2,382	52.9%	0.030	2,085	55.2%	2,082	55.2%	0.2
α glucosidase inhibitors	92	1.0%	69	1.5%	0.0065	44	1.2%	53	1.4%	2.1
Fixed dose therapies	2,293	24.9%	916	20.4%	< 0.0001	854	22.6%	840	22.3%	0.9
Cardiovascular	8,493	92.3%	4,111	91.4%	0.049	3,470	91.9%	3,463	91.8%	0.7
Antihyperlipidemics	6,526	71.0%	3,074	68.3%	0.0016	2,648	70.2%	2,638	69.9%	0.6
Antihypertensives	7,693	83.6%	3,814	84.8%	0.091	3,183	84.3%	3,187	84.4%	0.3
Other	3,164	34.4%	1,308	29.1%	< 0.0001	1,089	28.9%	1,106	29.3%	1.0
Antidepressants	2,890	31.4%	1,138	25.3%	< 0.0001	952	25.2%	978	25.9%	1.6
Antiobesity	82	0.9%	10	0.2%	< 0.0001	9	0.2%	10	0.3%	0.5
Antiemetics/antinausea	429	4.7%	260	5.8%	0.0051	202	5.4%	203	5.4%	0.1

^1^ Post-match balance was assessed by evaluating the standardized difference (std. diff.). All standardized differences were less than 2.5, except for mean age, which was 7.8.

Categorical variables were summarized by frequency and percentages. Continuous variables were reported by mean and standard deviation. Differences between treatments were tested for statistical significance using chi-square tests for categorical variables and t-tests or Wilcoxon rank tests for continuous variables. Kaplan-Meier plots were constructed to illustrate the probability of treatment modification for overall as well as each of the three modification measures in the post index period. Log-rank statistics and survival analysis were performed to assess the significance of the difference between treatments.

The data used for this study was based on an existing administrative health insurance claims database. There was no interaction with any subjects and the database does not include any individually identifiable data (e.g. does not include names, addresses, social security or medical record numbers, or any other obvious identifiers). The data is not developed or reported in a way that subjects can be identified, directly or through identifiers linked to subjects. According to information provided by the Office for Human Research Protections, this type of data is exempt for an IRB review under the requirements of the US Department of Health and Human Services regulations 45 CFR part 46. We acknowledge that many academic and medical institutions may have local IRBs that mandate that retrospective database studies apply for an IRB exemption; in this case there was no such mandate.

## Results

### Demographic and clinical characteristics

A total of 9,197 exenatide and 4,499 glargine patients met all inclusion and exclusion criteria in the pre-index period, prior to propensity score matching (Table 1). Propensity score matching resulted in a total of 3,774 matched pairs in the pre-index period. Baseline patient characteristics, including age, gender, health plan, clinical and resource use variables were balanced between the 2 cohorts after matching, with post-match standardized differences of less than 2.5 for all measures except age. Mean age in the exenatide cohort was 57.0 (standard deviation (SD = 10.8) years, compared to 57.8 (SD = 12.0) years in the glargine cohort (standardized difference = 7.8). Pre- and post-match descriptive measures are reported in Table 1.

### Treatment modification

**Figure 1:**
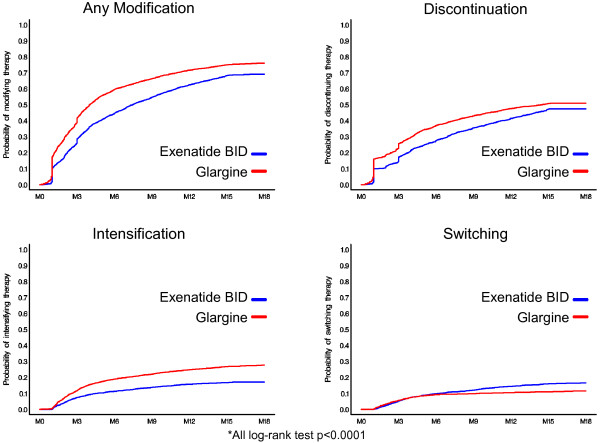
Treatment modification: Kaplan-Meier curves.

Compared to exenatide-treated patients, glargine-treated patients had lower treatment durability, demonstrated by higher rates of treatment modification and shorter time to treatment modification. Glargine-treated patients were 33% more likely to modify treatment [hazard ratio (HR) = 1.33, p < 0.0001]. Compared to exenatide-treated patients, a significantly higher percentage of glargine-treated patients experienced treatment modification in the 18 months post-index period (Table 2 and Figure 1). By 18 months, treatment modification was observed in 76.0% of the glargine cohort vs. 69.1% of the exenatide cohort (p < 0.0001). Patients initiating exenatide, were on their treatment longer before any modification compared to glargine-treated patients (159 vs. 123 days, p < 0.0001) indicating longer treatment durability.

**Table 2:** Treatment modification: frequency and timing

**Modification**	**Patients treated with**				**P value**
		**Exenatide BID**		**Insulin glargine**	
		N = 3,642*		N = 3,642*	
	**N/Mean**	**%/SD**	**N/Mean**	**%/SD**	
**Any type of treatment modification (Discontinuation, switching, or intensification)**					
By day 180 of follow-up	1,629	44.7%	2,169	59.6%	< 0.0001
By day 365 of follow-up	2,284	62.7%	2,616	71.8%	< 0.0001
By day 545 of follow-up	2,515	69.1%	2,768	76.0%	< 0.0001
Mean days to modification†	159	123	123	112	< 0.0001
**Intensification**					
By day 180 of follow-up	394	10.8%	660	18.1%	< 0.0001
By day 365 of follow-up	538	14.8%	856	23.5%	< 0.0001
By day 545 of follow-up	580	15.9%	947	26.0%	< 0.0001
Mean days to intensification†	150	117	154	128	0.541
**Discontinuation**					
By day 180 of follow-up	902	24.8%	1,163	31.9%	< 0.0001
By day 365 of follow-up	1,264	34.7%	1,396	38.3%	0.0013
By day 545 of follow-up	1,394	38.3%	1,456	40.0%	0.14
Mean days to discontinuation†	156	124	111	105	< 0.0001
**Switching**					
By day 180 of follow-up	333	9.1%	304	8.3%	0.23
By day 365 of follow-up	482	13.2%	340	9.3%	< 0.0001
By day 545 of follow-up	541	14.9%	365	10.0%	< 0.0001
Mean days to switching†	177	125	125	112	< 0.0001

* Patients with incomplete or invalid data on pharmacy claims were dropped; matched controls for these patients were also dropped.

† Mean days were calculated among patients with the specified modification.

Glargine-treated patients were 72% more likely to intensify therapy (HR = 1.72, p < 0.0001), with 26% of the glargine cohort vs. 15.9% of the exenatide cohort (p < 0.0001) having intensified by 18 months. The average time until treatment intensification was similar between exenatide and glargine patients (150 days vs. 154 days, p = 0.541). Similarly, there was a 25% greater likelihood of discontinuation of therapy in the glargine vs. exenatide cohorts (HR = 1.25, p < 0.0001). The average time until discontinuation was 156 days for exenatide-treated patients and 111 days for glargine-treated patients (p < 0.0001). Switching was the only treatment modification less likely to occur with glargine relative to exenatide-treated patients (HR = 0.71, p < 0.0001). However, among patients who did switch therapies, the average time until switching treatments was longer for exenatide-treated patients (177 days) compared to insulin glargine-treated patients (125 days, p < 0.0001).

Within the exenatide cohort, the most commonly used drug for intensification was biguanides (34.3% of patients with intensification), followed by sulfonylureas (18.3%) (Table 3). Glargine was used in only 8.3% of patients who initiated exenatide. Within the glargine cohort, intensification was most often accomplished by increasing the glargine dose (28.0%), followed by use of non-glargine insulin (21.1%). Intensification with exenatide was observed in only 5.9% of patients who initiated with glargine. Exenatide-treated patients who switched most often switched to sitagliptin (27.0%) or glargine (20.9%), whereas glargine-treated patients switched to a non-glargine insulin (27.1%), sulfonylureas (13.7%), biguanides (13.4%), or exenatide (13.2%).

**Table 3:** Treatment modification: specific drugs used in switching and intensification

**Drug**	**Patients treated with**			
		**Exenatide BID**		**Insulin glargine**
		N = 3,642*		N = 3,642*
	**N**	**%†**	**N**	**%†**
**Modification: Intensification**	580	100%	947	100%
Exenatide BID	0	0.0%	56	5.9%
Insulin glargine	48	8.3%	265	28.0%
Dose increased at least 100%			92	9.7%
Other insulin	28	4.8%	200	21.1%
Pre-mixed	2	0.3%	13	1.4%
Bolus	3	0.5%	184	19.4%
Basal (other than glargine)	23	4.0%	3	0.3%
Oral				
Sitagliptin	41	7.1%	65	6.9%
α glucosidase inhibitors	1	0.2%	1	0.1%
Thiazolidinediones	67	11.6%	85	9.0%
Meglitinides	14	2.4%	24	2.5%
Sulfonylureas	106	18.3%	78	8.2%
Biguanides	199	34.3%	114	12.0%
Fixed dose therapies	76	13.1%	54	5.7%
Other injectable/pramlintide	0	0.0%	5	0.5%
**Modification: switching**	541	100%	365	100%
Exenatide BID	0	0.0%	48	13.2%
Insulin glargine	113	20.9%	0	0.0%
Other insulin	65	12.0%	99	27.1%
Pre-mixed	17	3.1%	55	15.1%
Bolus	15	2.8%	23	6.3%
Basal (other than glargine)	33	6.1%	21	5.8%
Oral				
Sitagliptin	146	27.0%	31	8.5%
α glucosidase inhibitors	2	0.4%	0	0.0%
Thiazolidinediones	39	7.2%	46	12.6%
Meglitinides	8	1.5%	9	2.5%
Sulfonylureas	45	8.3%	50	13.7%
Biguanides	69	12.8%	49	13.4%
Fixed dose therapies	52	9.6%	33	9.0%
Other injectable/Pramlintide	2	0.4%	0	0.0%

* Patients with incomplete or invalid data on pharmacy claims were dropped; matched controls for these patients were also dropped.

† Percentages were calculated among patients with the specified modification.

## Discussion

There is little information on the ‘real-world’ medication utilization patterns for patients with type 2 diabetes initiating exenatide BID or glargine. The strength of this study is that it provides the ‘real-world’ data on physician prescribing behavior and patient medication use behavior over 18 months follow-up period in usual care settings. These observations used robust statistical approaches including propensity analyses to insure that the groups being compared were similar at baseline. In the current retrospective observational study, exenatide-treated patients had greater treatment durability as measured by a lower probability of treatment modification and a longer time to treatment modification. Glargine-treated patients were 33% more likely to modify (discontinue, intensify or switch) their treatment compared to exenatide-treated patients over the 18-month post-index period showing a consistent and steady difference, favorable to exenatide. The findings of this study are significant given the ongoing challenges associated with managing patients on therapies such as insulin or exenatide for achieving and maintaining glycemic control [10,11].

Treatment management for diabetes is often suboptimal, characterized by high rates of discontinuation, non-adherence, and lack of treatment modification [12], all of which are potential indicators of inadequate disease management including suboptimal glycemic control. Lower rates of persistence with insulin among new users are well-established; for example Cooke et al. reported that only 28.7% of new insulin users were persistent at 12 months [10]. In addition, a review article by Stephens et al. reported that better treatment persistence was associated with lower rates of diabetes-related complications, and also with reduced healthcare costs [13].

In the current study, exenatide-treated patients were less likely to discontinue or intensify treatment compared to glargine-treated patients. This is consistent with previously published reports by Fabunmi et al. [11] and Segal et al. [14]. Fabunmi et al. found that, compared to patients treated with glargine, exenatide-treated patients had a higher medication adherence (68.4% vs. 57.9%, p < 0.001), were more likely to have an adherence of at least 80% (46.9% vs. 29.4%, p < 0.001), and less likely to have either a 60 or 90-day gap in therapy (p < 0.001).

This current study did not examine reasons for treatment modification and there are multiple hypotheses as to why responses to or treatment patterns with these therapies might differ. For example, weight gain and hypoglycemia have been reported as significant reasons for discontinuation/switching of insulin therapy among patients with type 2 diabetes [15]. By contrast, exenatide has been shown to reduce patient weight [16-18], without increasing the risk of hypoglycemia common to several other glucose-lowering therapies [19]. In addition, the higher discontinuation rate without switching to another medication in glargine-treated patients could be due to tolerance issues or poor compliance; alternatively, this could be due to improved pancreatic beta cell function. Other potential reasons for treatment modification could be due to factors unrelated to improved glycemic control, such as cost or convenience. In addition, exenatide is a fixed dose therapy while glargine requires dose adjustment. Most patients who initiate glargine with low dose of 10 units per day would require an increase in dose to reach to the average dose of 30-40 units per day; this could be another reason that may influence treatment modification. In order to reduce this bias, the dose increase of 100% or more is considered as treatment modification for glargine patients. This study also found that glargine-treated patients were less likely to switch treatments than exenatide-treated patients (HR:0.71, p < 0.0001), which could be due, in part, to poorer tolerability of exenatide compared to insulin glargine. However, the exact reasons for treatment modifications cannot be identified with certainty from the current study and claims database.

Like any retrospective database analyses, this study has some limitations. First, the database comprised patients with commercial insurance coverage and results may not be generalizable to other patient populations. Second, clinical measures of glycemic control such as HbA1c or hypoglycemia and disease duration or severity are not available in this dataset. In the absence of HbA1c, diabetes-related complications, comorbidities and medication use in the pre-index period were used as a proxy to control for severity of disease in propensity score matching. Other variables impacting diabetes control, such as duration of diabetes, body weight/body mass index and adherence with life style modifications were not available in the database. Third, treatment modification measures were based on the presence or absence of prescription claims. These measures do not provide record of when or if medications were actually used; as such, this is an analysis of prescription filling patterns and not necessarily patient medication utilization behavior. Similarly, actual dose of insulin prescribed was not recorded in the claims database. Insulin glargine dose was calculated based upon insulin units dispensed and prescription refill dates, potentially leading to an overestimation of the quantity of insulin used by patients. Treatment intensification due to increased glargine dose, therefore, was defined as a 100% dose increase from one claim to the next in order to compensate for a potential overestimation of daily insulin doses. This approach was chosen to help insure that insulin glargine-treated patients who refilled their prescriptions early were not misclassified as patients intensifying treatment. This is the most conservative way of defining dose escalation for glargine in order to reduce potential bias towards exenatide. Fourth, while propensity score matching was used to reduce potential selection bias, and create two comparable patient groups, it does not control for potential unmeasured confounding variables. Also, the study only followed patients for 18 months following treatment initiation so adverse events (and related treatment modification) occurring after 18 months are not captured in this current analysis; it is unclear if longer term tolerability issues would have a differential impact on insulin glargine and exenatide. Limited information is available about the healthcare professionals that patients interact with, including prescribing physician speciality, educational programs, or medication therapy management initiatives often conducted by pharmacists or diabetes educators. This current analysis could not assess the presence of these resources or if access to them was different by study cohort. Lastly, it is important to note that the observational nature of the study design does not permit causal inferences.

## Conclusions

In conclusion, this study provides ‘real-world’ data showing significant differences in treatment modification rates for exenatide BID and insulin glargine, indicating potential for longer treatment durability for patients initiating exenatide BID. Future research should also examine the basis for the better medication utilization patterns associated with exenatide BID and whether those patterns translate into better clinical and economic outcomes.

## Competing interests

The authors have no competing interests or competing interest to report. Thomson Reuters (MB, BJ, RF, GL) was awarded a research contract for the conduct of this study from Eli Lilly and Company (MP, BJH). At the time of the study, MP and BJH were both full-time employees and stockholders of Eli Lilly and Company who funded the study and marketed exenatide with an Alliance partner, Amylin Pharmaceuticals, Inc. Exenatide is now solely marketed in the US by Amylin Pharmaceuticals, Inc.

## Authors’ contributions

All of the authors contributed to the study design, data interpretation, and discussion of study results. All authors approve of this manuscript and were active participants throughout the life of the study. MB was the principal investigator for the study. MP and BH developed the study concept and research questions. RF, GL, and BJ constructed the analytic file used for the study, operationalizing study concepts and linking them to specific research questions.

## Pre-publication history

The pre-publication history for this paper can be accessed here:

http://www.biomedcentral.com/1472-6823/13/20/prepub
